# Estimation of the Prevalence of Undiagnosed and Diagnosed HIV in an Urban Emergency Department

**DOI:** 10.1371/journal.pone.0027701

**Published:** 2011-11-16

**Authors:** William M. Reichmann, Rochelle P. Walensky, Amy Case, Anna Novais, Christian Arbelaez, Jeffrey N. Katz, Elena Losina

**Affiliations:** 1 Department of Orthopedic Surgery, Brigham and Women's Hospital, Boston, Massachusetts, United States of America; 2 Department of Biostatistics, Boston University School of Public Health, Boston, Massachusetts, United States of America; 3 Center for AIDS Research, Harvard Medical School, Boston, Massachusetts, United States of America; 4 Division of Infectious Disease, Massachusetts General Hospital, Boston, Massachusetts, United States of America; 5 Division of General Medicine, Massachusetts General Hospital, Boston, Massachusetts, United States of America; 6 Department of Emergency Medicine, Brigham and Women's Hospital, Boston, Massachusetts, United States of America; 7 Division of Infectious Disease, Brigham and Women's Hospital, Boston, Massachusetts, United States of America; 8 Division of Rheumatology, Immunology and Allergy, Brigham and Women's Hospital, Boston, Massachusetts, United States of America; 9 Department of Epidemiology, Harvard School of Public Health; Boston, Massachusetts, United States of America; Institut National de la Santé et de la Recherche Médicale, France

## Abstract

**Objective:**

To estimate the prevalence of undiagnosed HIV, the prevalence of diagnosed HIV, and proportion of HIV that is undiagnosed in populations with similar demographics as the Universal Screening for HIV in the Emergency Room (USHER) Trial and the Brigham and Women's Hospital (BWH) Emergency Department (ED) in Boston, MA. We also sought to estimate these quantities within demographic and risk behavior subgroups.

**Method:**

We used data from the USHER Trial, which was a randomized clinical trial of HIV screening conducted in the BWH ED. Since eligible participants were HIV-free at time of enrollment, we were able to calculate the prevalence of undiagnosed HIV. We used data from the Massachusetts Department of Public Health (MA/DPH) to estimate the prevalence of diagnosed HIV since the MA/DPH records the number of persons within MA who are HIV-positive. We calculated the proportion of HIV that is undiagnosed using these estimates of the prevalence of undiagnosed and diagnosed HIV. Estimates were stratified by age, sex, race/ethnicity, history of testing, and risk behaviors.

**Results:**

The overall expected prevalence of diagnosed HIV in a population similar to those presenting to the BWH ED was 0.71% (95% CI: 0.63%, 0.78%). The prevalence of undiagnosed HIV was estimated at 0.22% (95% CI: 0.10%, 0.42%) and resultant overall prevalence was 0.93%. The proportion of HIV-infection that is undiagnosed in this ED-based setting was estimated to be 23.7% (95% CI: 11.6%, 34.9%) of total HIV-infections.

**Conclusions:**

Despite different methodology, our estimate of the proportion of HIV that is undiagnosed in an ED-setting was similar to previous estimates based on national surveillance data. Universal routine testing programs in EDs should use these data to help plan their yield of HIV detection.

## Introduction

Among academic Emergency Departments (EDs) surveyed between December 2006 and March 2007, 13% (13/102) had instituted routine HIV screening policies in response to the 2006 Centers for Disease Control and Prevention (CDC) revised guidelines [1], [2]. The number of new cases identified by such routine testing programs depends greatly on the prevalence of undiagnosed HIV in these settings. The estimated proportion of HIV infection that remains undiagnosed in the United States decreased from 25% in 2000 [3] to 21% in 2006 [4] and to 20% in 2008 [5]. One possible explanation for this downward trend could be attributed to wide implementation of universal screening efforts. As universal HIV screening becomes more frequently implemented and the prevalence of undiagnosed HIV becomes less common, the proportion of HIV that is undiagnosed decreases. Older ED studies (1987–1990) throughout the nation have reported much higher estimates for the percentage of HIV that is undiagnosed, ranging from 49%–77% [6]–[10]. Studies from the mid-1990 s reported estimates of the percentage of HIV that is undiagnosed to be in line with more current CDC estimates in the US (range 20–28%) [11]–[13]. The most recent study, which was conducted by Clauss and colleagues in 2007, estimated that the proportion of HIV that was undiagnosed was 28.9% [14].

An accurate estimate of the current prevalence of undiagnosed HIV is critical to projecting the value of HIV screening programs. This paper provides a clear description of how we derived these estimates so that other investigators may apply these methods to their setting. This paper aims to report the overall prevalence of undiagnosed HIV-infection within specific demographic groups in the Brigham and Women's Hospital (BWH) ED from 2007–2009, during the Universal Screening for HIV in the Emergency Room (USHER) Trial. Using these estimates of the prevalence of undiagnosed HIV-infection, we also estimate the proportion of HIV infection that is undiagnosed in patients similar to those in the USHER study and the BWH ED with respect to age, race/ethnicity, sex, and risk behaviors.

## Methods

### Ethics statement

The Brigham and Women's Hospital institutional review board approved the study. Written informed consent was obtained from all participants in the USHER Trial.

### Data sources and elements

This analysis was conducted within the context of the USHER Trial. To be eligible for participation in the USHER Trial, ED patients had to be: 1) between the ages of 18 and 75; 2) English- or Spanish-speaking; and 3) not known to be HIV-infected. Patients with an emergency severity index (ESI) score of 1 or 2 (on a scale of 1 to 5, with 1 being most severe) needed written authorization from the attending ED physician to be considered eligible [15]–[17]. A complete set of inclusion and exclusion criteria for USHER Trial are presented elsewhere [18]. In addition to data from the USHER Trial, we used data from the BWH ED, the Massachusetts Department of Public Health (MA/DPH) surveillance program [19], and the US Census Bureau [20].

#### USHER Trial data

Demographic data (age, race/ethnicity, and sex) were collected at the time of enrollment. For the purposes of this analysis, we dichotomized age as those less than 45 years and those 45 years or older. We chose 45 years because it corresponded to the median age among newly identified cases of HIV-infection in the USHER Trial. We categorized race/ethnicity into four groups; 1) non-Hispanic white, 2) non-Hispanic black, 3) Hispanic, and 4) other. Enrolled participants were also asked to complete an 86-item survey. Details of the survey have been published elsewhere [21]. From this survey, we used data on self-reported history of testing and risk behaviors. History of testing was categorized into four groups; 1) previously tested/no date reported, 2) previously tested/within the last 5 years, 3) previously tested/5 or more years ago, 4) missing history of testing information. We chose a five year threshold because we hypothesized that the participants' ability to recall with any accuracy beyond five years was limited and that there have been secular changes in HIV testing over the past five years. Participants were classified as having a sexual risk behavior if they reported one of the following: 1) greater than one sexual partner during the last 12 months, 2) reported condom use as less than always, or 3) self-report of having sexual contact with partners who are known to have HIV, have been incarcerated, or who use injectable illicit drugs. Alcohol risk behavior was assessed using the 10-item Alcohol Use Disorders Identification Test (AUDIT) [22]. A score of 8 or higher was used to classify those as having the alcohol risk behavior. Data on use of 10 illicit drugs (heroin, cocaine/crack, speed, oxycontin/other narcotics, poppers, marijuana, crystal methamphetamine, LSD, ecstasy, and other) were collected. Participants were classified as having the illicit drug use risk behavior if they reported having used one of the drugs at least occasionally or two of the drugs once. We analyzed the missing data patterns by calculating the proportion missing for those variables among those that were and were not tested in the USHER Trial. If the distributions of missing values were similar for those who were and were not tested then we can consider the data to be missing at random (MAR). Data that are MAR yield point estimates that are not biased when using standard statistical methodology. However, precision may be affected due to the decrease in sample size [23].

#### BWH ED data

We obtained demographic data (age, race/ethnicity, and sex) for all patients who visited the ED between January 1, 2008 and May 31, 2009. Age and race data were categorized similarly to that of the USHER Trial demographic data.

#### MA/DPH HIV Surveillance data

The number of people living with HIV or AIDS in 2008 was obtained from the MA/DPH surveillance program. The MA/DPH surveillance program classified the number of cases of HIV-infection by age (18–24, 25–34, 35–44, 45–54, 55–64, and 65+), sex, and race/ethnicity (non-Hispanic white, non-Hispanic black, Hispanic, and other).

#### US Census Bureau data

The number of people residing in MA in 2008 stratified by the same age, sex, and race/ethnicity groups provided by the MA/DPH surveillance program was obtained from published estimates from the US Census Bureau [20].

### Data Analysis

#### Estimation of the prevalence of undiagnosed HIV-infection

The prevalence of undiagnosed HIV-infection was calculated as the number of new HIV diagnoses in the USHER Trial divided by the number of trial participants tested. This quantity is an estimate of the prevalence of undiagnosed HIV because those who self-reported prior HIV diagnoses were not eligible for enrollment in the USHER Trial. We estimated the overall prevalence of undiagnosed HIV-infection and for specific demographic and risk subgroups in the USHER Trial. Clopper-Pearson 95% confidence intervals, which is an exact interval based on the binomial distribution, were constructed for all estimates [24].

#### Estimation of the expected prevalence of diagnosed HIV-infection

We estimated the expected prevalence of diagnosed HIV-infection for the BWH ED by multiplying the mean prevalence for each subgroup in the MA/DPH surveillance program by the proportion per subgroup among all patients presenting to the BWH ED. This step-by-step process was carried out as follows:

1. The prevalence of diagnosed HIV-infection was calculated by age, sex, and race/ethnicity for the state of MA. To do so, the number of people known to be living with a diagnosis of HIV/AIDS from the MA/DPH surveillance program was divided by the number of people residing in MA from published estimates from the US Census Bureau (data not shown) [20].
2. We calculated weights for each age, sex, and race/ethnicity combination in the BWH ED by dividing the number of patients in each stratum by the total number of patients presenting to the BWH ED.
3. Lastly, the age, sex, and race/ethnicity specific prevalences in MA were multiplied by corresponding weights in the BWH ED and then summed to obtain the expected weighted prevalence of diagnosed HIV-infection in the BWH ED.

To estimate the prevalence of diagnosed HIV-infection for each level of a variable, the weights had to sum to one. For example, the estimated prevalence of diagnosed HIV among those 18–44 years old, male, and non-Hispanic White was 0.26%. This sub-population represented 9.7% of the overall population in the BWH ED. These two quantities were multiplied together and the process was repeated for all age, sex, and race/ethnicity groupings. The sum of these values serves as an estimated overall expected prevalence of diagnosed HIV in a population similar to the BWH ED. To estimate the prevalence of diagnosed HIV-infection for those ages 18–44, the weights were calculated among those 18–44 as the number of patients within each combination of sex and race/ethnicity divided by the total number of people ages 18–44. These weights were then multiplied by the corresponding prevalence of HIV among those 18–44 within each sex and race/ethnicity combination and summed to obtain the weighted prevalence of HIV for those ages 18–44. The calculation was similar for the expected prevalence of diagnosed HIV-infection by race/ethnicity and sex.

We also calculated the prevalence of diagnosed HIV-infection by history of testing and risk behaviors. To do this, we used the method above within each subgroup for history of testing and risk behavior. For example we used the distribution of age, race/ethnicity, and sex in the USHER Trial among those who were previously tested in the last five years as the weights for estimating the expected prevalence of diagnosed HIV-infection among those had been previously tested in the previous five years. The sampling distribution of the expected prevalence of diagnosed HIV-infection is not known because it is a weighted prevalence. Thus, we used simulations to construct empirical 95% confidence intervals for all estimates (see Supporting Information Appendix S1) [25].

#### Estimation of percentage of HIV that is undiagnosed

The percentage of HIV that is undiagnosed for persons similar to those presenting to the BWH ED was calculated by dividing the prevalence of undiagnosed HIV-infection by the sum of the prevalence of undiagnosed HIV-infection and the estimated prevalence of diagnosed HIV-infection. The formula is shown below where p_u_ represents the prevalence of undiagnosed HIV-infection and p_d_ represents the prevalence of diagnosed HIV-infection.
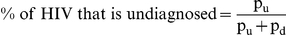
Similar to the expected prevalence of diagnosed HIV-infection, the percentage of HIV that is undiagnosed does not follow a known distribution. Thus, empirical 95% confidence intervals were constructed for all estimates (see Supporting Information Appendix S1 for technical detail regarding construction of empirical 95% confidence interval) [25].

#### Estimation of the ratio of undiagnosed HIV to diagnosed HIV

Lastly, we also estimated the ratio of undiagnosed HIV to diagnosed HIV. This was done by dividing the estimated prevalence of undiagnosed HIV by the estimated prevalence of diagnosed HIV. This metric will be useful in situations when only the prevalence of diagnosed HIV is known as the prevalence of undiagnosed HIV can be estimated by simply multiplying this ratio by the known prevalence of diagnosed HIV infection in a particular setting.

## Results

### Demographics of USHER Trial participants who were tested for HIV

A total of 4,056 participants were HIV tested in the USHER Trial. Of these, 2,954 (75%) were between the ages of 18–44; 2,604 (65%) were female; 1,190 (29%) were non-Hispanic white; 915 (23%) were non-Hispanic black; 1,461 (36%) were Hispanic, and 490 (12%) were other (Table 1). The distribution of the history of testing and HIV risk behaviors are also shown in Table 1.

**Table 1:** Prevalence of undiagnosed HIV in the USHER Trial for the overall sample and by demographic and risk factors.

	Number tested (%)	Number HIV positive	Prevalence of undiagnosed HIV(95% CI)
Overall	4,056	9	0.22% (0.10, 0.42)
Age			
18–44 years old	2,954 (75%)	4	0.14% (0.04, 0.35)
45–75 years old	987 (25%)	5	0.51% (0.16, 1.18)
Sex			
Male	1,428 (35%)	8	0.56% (0.24, 1.10)
Female	2,604 (65%)	1	0.04% (0.00, 0.21)
Age and Sex			
18–44 years old			
Male	1,010 (26%)	4	0.40% (0.11, 1.01)
Female	1,929 (49%)	0	0.00% (0.00, 0.19)
45–75 years old			
Male	377 (10%)	4	1.06% (0.29, 2.69)
Female	603 (15%)	1	0.17% (0.00, 0.92)
Race/ethnicity			
Non-Hispanic White	1,190 (29%)	2	0.17% (0.02, 0.61)
Non-Hispanic Black	915 (23%)	2	0.22% (0.03, 0.79)
Hispanic	1,461 (36%)	3	0.21% (0.04, 0.60)
Other	490 (12%)	2	0.41% (0.05, 1.47)
History of Testing			
Previously tested/no date	376 (9%)	1	0.27% (0.01, 1.47)
Previously tested/<5 years	1,034 (25%)	1	0.10% (0.00, 0.54)
Previously tested/5+years or never tested	1,172 (29%)	4	0.34% (0.09, 0.87)
Missing history of testing information	1,474 (36%)	3	0.20% (0.04, 0.59)
Sexual Risk*			
Yes	2,187 (54%)	4	0.18% (0.05, 0.47)
No	310 (8%)	0	0.00% (0.00, 1.18)
Missing	1,559 (38%)	5	0.32% (0.10, 0.75)
Alcohol risk**			
Yes	365 (9%)	1	0.27% (0.01, 1.52)
No	1,941 (48%)	4	0.21% (0.06, 0.53)
Missing	1,750 (43%)	4	0.23% (0.06, 0.58)
Drug Risk***			
Yes	1,024 (25%)	3	0.29% (0.06, 0.85)
No	1,293 (32%)	1	0.08% (0.00, 0.43)
Missing	1,739 (43%)	5	0.29% (0.09, 0.67)

*Participants who had missing data for three or more of the items related to sexual risk and not reporting risk behavior on other factors were classified as ‘missing’.

**A score of 8 or higher is considered as having the alcohol risk behavior. If answers were provided to fewer than 5 questions, the alcohol risk behavior was noted as ‘missing’.

***If answers to at least 3 drug-related questions were missing and no other drug-related behavior was reported, the drug behavior was marked ‘missing’.

### Missing data patterns of history of testing and risk behaviors

We compared the proportion of missing data for certain parameters of interest between those tested in the USHER Trial and those not tested. We found that the proportion missing data for history of testing was similar for those tested in the USHER Trial (36%) compared to those who were not tested in the USHER Trial (39%). The proportion of missing data for the risk behaviors was slightly higher among those who were not tested in the USHER Trial. For the alcohol risk behavior, 48% of those not tested were missing compared to 41% of those tested in the USHER Trial. For the drug risk behavior, 50% of those not tested were missing compared to 43% of those who were tested in the USHER Trial. Lastly, for the sexual risk behavior, 44% of those not tested were missing compared to 38% of those who were tested in the USHER Trial.

### Prevalence of undiagnosed HIV in the USHER Trial

The USHER Trial identified 9 new cases of HIV-infection for an undiagnosed prevalence of 0.22% (95% CI: 0.10%, 0.42%). The prevalence of undiagnosed HIV was 0.51% (95%CI: 0.16%, 1.18%) in those age 45–75 years compared to 0.14% (95% CI: 0.04%, 0.35%) in those 18–44 years of age. The prevalence of undiagnosed HIV ranged from 0.04% (95% CI: 0.00%, 0.21%) in females to 0.56% (95% CI: 0.24%, 1.10%) in males. The prevalence of undiagnosed HIV was similar across race/ethnicity groups. Those who were previously tested in the last 5 years had an estimated prevalence of undiagnosed HIV of 0.10% (95% CI: 0.00%, 0.54%) compared to 0.34% (95% CI: 0.09%, 0.87%) for those who were previously tested five or more years ago or never tested. Estimates of the prevalence of undiagnosed HIV within the trial are shown in Table 1 for all subgroups.

### Estimated percentage of HIV that is undiagnosed based on BWH ED demographics

The overall expected prevalence of diagnosed HIV in a population similar to those presenting to the BWH ED was estimated to be 0.71% (95% CI: 0.63%, 0.78%). This estimate of anticipated diagnosed HIV infection combined with the estimate of the prevalence of undiagnosed HIV (0.22%) yielded an overall prevalence of 0.93%.To obtain the proportion of HIV that is undiagnosed based on trial demographics, the prevalence of undiagnosed HIV (0.22%) was divided by the overall prevalence (0.93%); a projected 23.7% (95% CI: 11.6%, 34.9%) of all HIV infections were undiagnosed. The proportion of HIV-infection that was undiagnosed was estimated for demographic subgroups (Table 2). Among those 18–44 years old, 19.4% (95% CI: 5.1%, 33.8%) of HIV infection is estimated to be undiagnosed compared to 37.0% (95% CI: 10.3%, 54.2%) among those 45–75 years old. The estimated proportion of HIV that was undiagnosed was 37.1% (95% CI: 18.0%, 51.0%) for males and 6.8% (95% CI: 0.0%, 19.3%) for females. Those of other race had the highest estimated proportion of undiagnosed HIV (80.4%; 95% CI: 0.0%, 98.4%). The proportion of HIV that was undiagnosed among non-Hispanic whites, non-Hispanic blacks, and Hispanics was 48.6% (95% CI: 0.0%, 72.4%), 11.2% (95% CI: 0.0%, 23.6%), and 16.0% (95% CI: 0.0%, 30.6%), respectively.

**Table 2:** The expected prevalence of diagnosed HIV and percentage of HIV that is undiagnosed based on the age, race/ethnicity, and sex distributions in the BWH Emergency Department and prevalence of diagnosed HIV in the MA/DPH Surveillance Program.

	N (%)	Prevalence of diagnosed HIV(95% CI)	Percentage of HIV that is undiagnosed*(95% CI)	Ratio of undiagnosed to diagnosed HIV
Overall	48,599	0.71% (0.63, 0.78)	23.7% (11.6, 34.9)	0.31
Age				
18–44 years old	26,942 (55%)	0.58% (0.50, 0.68)	19.4% (5.1, 33.8)	0.24
45–75 years old	21,657 (45%)	0.87% (0.76, 1.00)	37.0% (10.3, 54.2)	0.59
Sex				
Male	19,284 (40%)	0.95% (0.81, 1.09)	37.1% (18.0, 51.0)	0.59
Female	29,315 (60%)	0.55% (0.46, 0.63)	6.8% (0.0, 19.3)	0.07
Age and Sex				
18–44 years old				
Male	9,780 (20%)	0.65% (0.49, 0.81)	38.1% (11.6, 58.0)	0.62
Female	17,162 (35%)	0.54% (0.43, 0.66)	0.0% (0.0, 10.1)	0.00
45–75 years old				
Male	9,504 (20%)	1.24% (1.02, 1.47)	46.1% (15.4, 63.9)	0.85
Female	12,153 (25%)	0.58% (0.44, 0.72)	22.7% (0.0, 48.7)	0.29
Race/ethnicity				
Non-Hispanic White	24,373 (50%)	0.18% (0.13, 0.23)	48.6% (0.0, 72.4)	0.94
Non-Hispanic Black	10,973 (23%)	1.74% (1.49, 2.00)	11.2% (0.0, 23.6)	0.13
Hispanic	9,558 (20%)	1.10% (0.90, 1.31)	16.0% (0.0, 30.6)	0.19
Other	3,695 (8%)	0.10% (0.00, 0.22)	80.4% (0.0, 98.4)	4.10

*Percentage of HIV that is undiagnosed is calculated using the estimates of prevalence of undiagnosed HIV from Table 1.

### Estimated percentage of HIV that is undiagnosed by history of testing and risk behavior

The proportion of undiagnosed HIV-infection for those who previously tested in the last 5 years was 11.9% (95% CI: 0.0%, 40.0%), while it was 35.8% (95% CI: 8.3%, 66.7%) who were tested 5 or more years ago or were never tested. The percentage of HIV that was undiagnosed among those reporting sexual risk behavior was 20.5% (95% CI: 3.8%, 42.1%), compared to 0.0% (95% CI: 0.0%, 100.0%) among those not reporting a sexual risk behavior. Those reporting an illicit drug risk behavior had proportion of HIV that was undiagnosed estimated at 31.5% (95% CI: 0.0%, 66.7%) compared to 10.1% (95% CI: 0.0%, 33.3%) in those who not report using illicit drugs. The proportion of HIV that is undiagnosed was similar by alcohol risk behavior group (Table 3).

**Table 3:** The expected prevalence of diagnosed HIV and percentage of HIV that is undiagnosed based on HIV history of testing and risk behavior in the USHER Trial and prevalence of diagnosed HIV in the MA/DPH Surveillance Program.

	Prevalence of diagnosed HIV (95% CI)	Percentage of HIV that is undiagnosed*(95% CI)	Ratio of undiagnosed to diagnosed HIV
History of Testing			
Previously tested/no date	0.78% (0.00, 1.86)	25.7% (0.0, 100.0)	0.35
Previously tested/<5 years	0.74% (0.29, 1.35)	11.9% (0.0, 40.0)	0.14
Previously tested/5+years or never tested	0.61% (0.26, 1.11)	35.8% (8.3, 66.7)	0.56
Missing history of testing information	0.89% (0.41, 1.42)	18.3% (0.0, 41.2)	0.22
Sexual Risk			
Yes	0.70% (0.37, 1.05)	20.5% (3.8, 42.1)	0.26
No	0.66% (0.00, 1.61)	0.0% (0.0, 100.0)	0.00
Missing	0.87% (0.38, 1.41)	26.9% (7.7, 50.0)	0.37
Alcohol risk			
Yes	0.60% (0.00, 1.37)	31.0% (0.0, 100.0)	0.45
No	0.68% (0.36, 1.08)	23.6% (5.3, 45.0)	0.31
Missing	0.88% (0.46, 1.37)	20.7% (5.0, 41.7)	0.26
Drug Risk			
Yes	0.63% (0.20, 1.17)	31.5% (0.0, 66.7)	0.46
No	0.71% (0.31, 1.24)	10.1% (0.0, 33.3)	0.11
Missing	0.88% (0.52, 1.32)	24.8% (6.3, 44.4)	0.33

*Percentage of HIV that is undiagnosed is calculated using the estimates of prevalence of undiagnosed HIV from Table 1.

#### Estimation of the ratio of undiagnosed HIV to diagnosed HIV

The ratio of undiagnosed HIV to diagnosed HIV in a population with demographic features similar to the BWH ED was 0.31. Estimates varied in a similar fashion as the estimated percentage of HIV that is undiagnosed. Estimates for the ratio of undiagnosed HIV to diagnosed HIV are shown in Tables 2 and 3.

## Discussion

We estimated the proportion of HIV-infection that is undiagnosed in the ED setting based on data from the USHER Trial and 2008 MA/DPH surveillance data. We found the overall proportion of HIV-infection that is undiagnosed to be 23.7% (95% CI: 11.6%, 34.9%) when applying the BWH ED demographic distribution. This value for the estimated proportion of HIV-infection that is undiagnosed is difficult to determine and is concordant with estimates from the CDC, though our method of calculation differs from the CDC's method [4], [5]. The CDC reports that 20% of all HIV is undiagnosed in the US as of 2008 [5]. Our findings are further supported by data from one Denver ED where a blinded seroprevalence screening for HIV reported no record of state-documented HIV infection for 20% of patients who tested positive for the HIV antibody [12]. While the study by Clauss et al. estimated the proportion of HIV that is undiagnosed to be slightly higher at 28.9%, their estimate falls within the 95% confidence interval of the overall estimate presented in this paper. In this study, they conducted a blinded seroprevalence study while linking demographic and clinical information to HIV status, which allowed them to classify patients as having been diagnosed or not. It is possible that previous diagnosis was misclassified if the patient was diagnosed at a different institution. This may lead to a small overestimation of the proportion of HIV that is undiagnosed in that study. In contrast, our study calculated an estimate of the prevalence of undiagnosed HIV-infection from a sample drawn directly from the BWH ED and coupled it with an estimate of the prevalence of diagnosed HIV-infection based on MA/DPH surveillance data weighted by the demographic distribution in the BWH ED [14].

In addition to the consistency of our results with others, despite different methodology, our study is unique in that we were able to calculate the proportion of undiagnosed HIV by specific subgroups within the ED setting. We found that those 45 years of age or older, men, non-Hispanic whites, and those who have not recently been tested have a higher proportion of HIV-infection that was undiagnosed among those with the disease. Our results stratified by these specific subgroups do not necessarily match the results provided by the CDC. According to the CDC, those 45 years of age or older and non-Hispanic white were less likely to be undiagnosed among those with HIV and the slight increase for men was much smaller than what we found. The CDC did not evaluate history of testing [4], [5]. We anticipate these results could be due to a number of factors. First, these subgroups are generally not suspected of being HIV-infected so they may not be offered HIV testing as frequently. This hypothesis would apply mostly to participants who were older and of non-Hispanic white race, with demographic characteristics traditionally less associated with HIV infection. Second, such persons may be more likely to refuse HIV testing in the ED setting. This theory is corroborated by other published USHER data that demonstrated those who were older and non-Hispanic white were more likely to decline the test offer [26]. Lastly, differences could also be attributed to the two different estimation methods. We used USHER Trial data supplemented with data from the MA/DPH Surveillance Program, while the CDC used an extended back calculation method [4], [5].

There were a small number of undiagnosed cases found in the USHER Trial, and our results could be due to uncertainty. Due to the generally low prevalence of HIV, the estimated confidence intervals are wide, especially for the estimation of the proportion of HIV that is undiagnosed by specific subgroups. We suggest that upper and lower confidence bounds should therefore be considered to define plausible ranges for decision making, health care planning and policy strategies. Because the goal was to estimate prevalence, we did not report any p-values. Instead, we used 95% confidence intervals as a measure of data uncertainty. We also had a high rate of missing data for history of testing and our assessment of risk behaviors. This was due to participants both refusing to fill out the survey and not completing an already started survey [27]. Analysis of our missing data patterns suggested that missing data on history of testing were missing at random, but that missing data on assessment of risk behaviors exhibited a slight imbalance between those who were and were not tested. Since such imbalance was relatively small, we do not anticipate it will lead to appreciable bias in our risk-stratified prevalence estimates [23].

This study has substantial implications for future study designs. As HIV testing becomes more prevalent in all settings, the proportion of HIV that is undiagnosed will continue to decrease. Because of this decline, future studies that rely on the overall prevalence (or diagnosed prevalence) of HIV in their specific population as basis for power and sample size calculations will greatly underestimate the number of subjects that need to be recruited into their study. Also of note is that the CDC recommends that routine screening programs should be performed in settings that report an undiagnosed prevalence that is greater than 0.1% [2]. This method of calculating the proportion of HIV that is undiagnosed can be implemented by other EDs that have implemented a screening program, provided they have the data on newly diagnosed HIV and an estimate of the prevalence of known HIV diagnoses for their ED. This can help inform ED administrations on the feasibility of implementing an HIV screening program.

A recent study by Lindsell et al. estimated the undiagnosed prevalence of HIV to be 0.05% in an urban academic ED. In the absence of data from their ED, they used zip code-specific case rates of new diagnoses from clinics in the surrounding area of the ED. They then multiplied these case rates by distribution of zip codes within the ED and summed them to obtain estimate of prevalence for the entire ED. The applicability of this method depends greatly on the availability of data from multiple clinics and treatment centers surrounding the ED. Even then, these clinics may not reach the scope of patients that will attend that particular ED [28].

We acknowledge that the prevalence estimates in our analyses were derived from a single high-volume center. To use our data to estimate undiagnosed prevalence in other settings, we suggest that distributions of demographic characteristics and risk factors in the population of interest should be combined with stratified estimates, obtained from the current analysis. A weighted average approach then will facilitate estimation of undiagnosed prevalence in the population of interest. Lastly, enrollment in the USHER Trial did not cover overnight times and missed some weekends. Thus, some HIV-infection may have been missed as these represent the times that those at highest risk of HIV-infection present to the ED.

Using data from an ED-based clinical trial, we found that 23.7% of all HIV infection is undiagnosed, a number similar to that reported by the CDC. While there is considerable variability in our estimates, our findings further note that the fraction of undiagnosed HIV infection likely varies by demographic factors and risk categories, including age, sex, race/ethnicity, history of testing, sexual risk, and illicit drug use. These results suggest further highlighting where targeted testing interventions may offer the highest yield.

## Supporting Information

Appendix S1 Supporting information.
(DOC)
