# Supplementary material for: Estimation of the Prevalence of Undiagnosed and Diagnosed HIV in an Urban Emergency Department
Source: PLoS One. 2011 Nov 16;6(11):e27701. doi: 10.1371/journal.pone.0027701 (PMC3218027; doi:10.1371/journal.pone.0027701)
Supplement: Appendix S1 — Supporting information. (DOC) [file pone.0027701.s001.doc]

**SUPPORTING INFORMATION**

*Construction of Empirical 95% Confidence Intervals*

Simulations were used to construct empirical 95% confidence intervals for the prevalence of diagnosed HIV-infection and for the percentage of HIV that is undiagnosed. The step-by-step process was performed as follows:

1. Pairs of samples were drawn at random. Sample 1 was used to estimate the prevalence of undiagnosed HIV. For sample 1, we assumed a binomial distribution and used the estimated prevalence of undiagnosed HIV to generate the number of undiagnosed HIV cases and the corresponding sample size of the USHER Trial. For example, when estimating for the entire population, the sample size was 4,056 and when estimating for those ages 18-44, the sample size was 2,954.
2. Sample 2 was used to estimate the prevalence of diagnosed HIV. Again, we assumed a binomial distribution and used the estimated prevalence of diagnosed HIV. When comparing to persons demographically similar to those presenting to the BWH ED, we used the sample size from the BWH ED. When comparing to persons demographically similar to those tested in the USHER Trial, we used the sample size from the USHER Trial.
3. The simulation was run 1,000 times and 1,000 estimates of the prevalence of undiagnosed and diagnosed HIV were calculated.
4. The empirical 95% confidence interval for the prevalence of diagnosed HIV was derived as the 2.5^th^ percentile (lower bound) and the 97.5^th^ percentile (upper bound) of the distribution of the 1,000 estimates obtained from the simulation [25].
5. Using the estimates obtained in step 3, we also calculated 1,000 estimates of the proportion of HIV that is undiagnosed.

Similar to step four, the empirical 95% confidence interval for the proportion of HIV that was diagnosed was derived as the 2.5^th^ percentile (lower bound) and the 97.5^th^ percentile (upper bound) of the distribution of the 1,000 estimates obtained from the simulation [25].
